# Simulative and Experimental Characterization of an Adaptive Astigmatic Membrane Mirror

**DOI:** 10.3390/mi12020156

**Published:** 2021-02-05

**Authors:** Ulrich Kallmann, Michael Lootze, Ulrich Mescheder

**Affiliations:** 1 Department of Business Admin & Engineering, Furtwangen University of Applied Science, D-78120 Furtwangen, Germany; 2 Institute for Microsystems Technology (IMST), Department of Mechanical & Medical Engineering, Furtwangen University of Applied Science, D-78120 Furtwangen, Germany; michael.lootze@hs-furtwangen.de (M.L.); mes@hs-furtwangen.de (U.M.); 3 Associated to the Faculty of Engineering, University of Freiburg, D-79110 Freiburg, Germany

**Keywords:** adaptive optics, optical simulation, physical optics, micro-optical electro-mechanical system (MOEMS), silicon-on-insulator (SOI), electrostatic actuation, active focusing, thin film stress, finite element method (FEM)

## Abstract

Adaptive optical (AO) components play an important role in numerous optical applications, from astronomical telescopes to microscope imaging systems. For most of these AO components, the induced wavefront correction, respectively added optical power, is based on a rotationally symmetric or segmented design of the AO component. In this work, we report on the design, fabrication, and characterization of a micro-electronic-mechanical system (MEMS) adaptive membrane mirror in the shape of a parabolic cylinder. In order to interpret the experimental characterization results correctly and provide a tool for future application development, this is accompanied by the setup of an optical simulation model. The characterization results showed a parabolically deformable membrane mirror with an aperture of 8 × 2 mm^2^ and an adaptive range for the optical power from 0.3 to 6.1 m^−1^ (dpt). The optical simulation model, using the Gaussian beamlet propagation method, was successfully validated by laser beam profile measurements taken in the optical characterization setup. This MEMS-based adaptive astigmatic membrane mirror, together with the accompanying simulation model, could be a key component for the rapid development of new optical systems, e.g., adaptive laser line generators.

## 1. Introduction

An increasing number of optical systems utilize adaptive optical (AO) components, which allow for an electronically controlled variation of the optical key parameters. These components range from surface curvature-changing liquid-filled membrane devices up to micro-electronic-mechanical system (MEMS)-based deformable mirrors [1,2].

For these systems, a widespread range of optical applications can be found. As a means for wavefront correction, they are, for example, used in astronomical telescopes [1], microscope imaging systems [3,4], and ophthalmic systems [5]. Piezo actuator-driven AO components [6] provide here wavefront correction over optical apertures of more than 20 mm. Furthermore, a segmented AO design allows to correct for several types of aberration, such as defocusing, comas, and astigmatism [7,8]. The induced deformation in the AO component typically can lead to a wavefront correction in the range of 10 to 20 waves (about 5–10 µm).

For applications which demand a higher adaptive range in optical power, liquid-filled polymer membrane lenses are commercially available [9]. Electronically tunable lenses with a clear aperture of 10 mm can achieve a variation in optical power of more than 10 m^−1^ (dpt) [10]. These lenses, in combination with fixed focus lenses, are used in machine vision applications, for example, requiring auto-focus for varying object distances. As another application example, an AO component based on the electrowetting principle has been demonstrated as a device for beam steering in laser processing or LIDAR systems [11].

In this article, we present and characterize a novel AO component, a MEMS-based adaptive astigmatic membrane (MAAM) mirror. Our MAAM mirror is designed to provide a variable parabolic profile in one mirror surface direction, while providing a flat profile in the perpendicular surface direction. In geometric terms, the mirror represents a concave parabolic cylinder surface. There are reports of parabolically deformable cylinder surfaces [12,13], but to our knowledge it is the first adaptive parabolic cylinder mirror realized with MEMS-technology. In [14], a system is presented where focusing of mid-infrared light with a fixed focal length is derived from a metasurface, and the focussed beam is then scanned in two directions by ±9° with an integrated MEMS scanner. However, in our approach, in situ beam shaping is achieved by the use of an MEMS deformable mirror in the form of a parabolic cylinder as an active focusing device.

**Figure 1:**
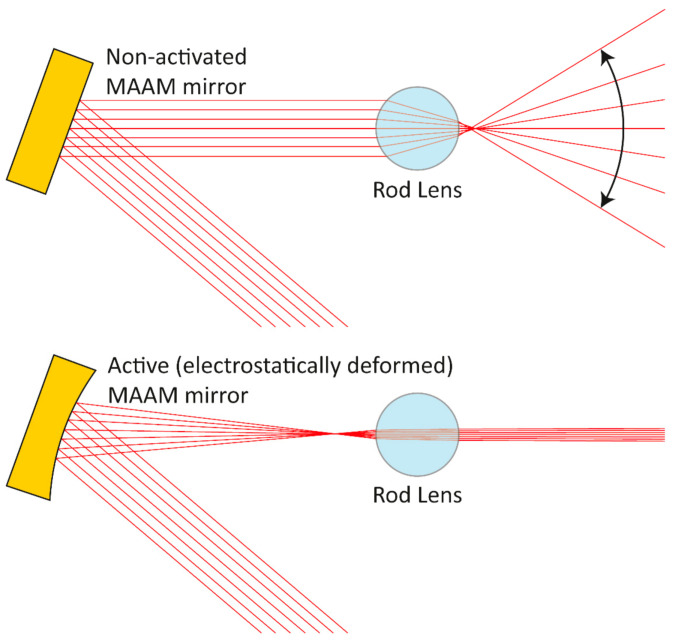
Use of the micro-electronic-mechanical system-based adaptive astigmatic membrane (MAAM) mirror in a laser line setup with a variable fan angle (schematic top view): with a non-activated flat MAAM mirror (**top**), the full aperture of the rod lens is illuminated and leads to a wide fan angle. Focussing (**bottom**) illuminates a smaller section of the rod lens, leading to a smaller fan angle.

For opening up a broad range of applications, the design aims at a variation in the optical power of at least 5 dpt, while providing, in its first prototype, a clear aperture of 8 mm × 2 mm. Possible applications for such an AO component lie in the generation of a variable focus in one beam direction for laser processing or in the generation of a laser line with a variable fan angle, as depicted in Figure 1.

In order to leverage future applications of the MAAM mirror, the characterization is accompanied by the development of an optical simulation model. A validated simulation model contributes significantly to reduced cost and development time for future applications. This approach, the extensive use of simulation methods in early development stages, is often referred to as simulation frontloading [15,16]. It has been successfully demonstrated in many engineering domains, e.g., in electronic circuit design and also in optical engineering for numerous incoherent imaging or illumination system design tasks [17].

In our proposed application cases, we deal with coherent free space light propagation. As can be seen, e.g., in Figure 1, these applications also include highly divergent light beams. Although a number of methods in the field of physical optics exist to model coherent optical systems [18,19], most of them are not suited to handle such divergent beam situations [20]. To provide a general simulation model, which also supports highly divergence beams, the Gaussian beamlet propagation (GBP) method for the simulation was used [21,22,23,24]. GBP has previously shown its potential as a simulation method for line lasers with highly divergent beam geometries [25]. In this article, it will be investigated whether this method can be transferred to a laser beam forming system, using the MAAM mirror.

## 2. Materials and Methods

In this section, we will start with the description of the electromechanical and optical properties of the MAAM mirror. The parameters described here are further used as input parameters for the coherent system simulation model. This is followed by the description of the experimental setup, which resembles all important optical and mechanical properties of the coherent system simulation model. Having established methods to obtain simulative and experimental data to characterize the MAAM mirror, an approach to compare these data is introduced.

### 2.1. Design, Fabrication, Electromechanical and Optical Properties of the MAAM Mirror

**Figure 2:**
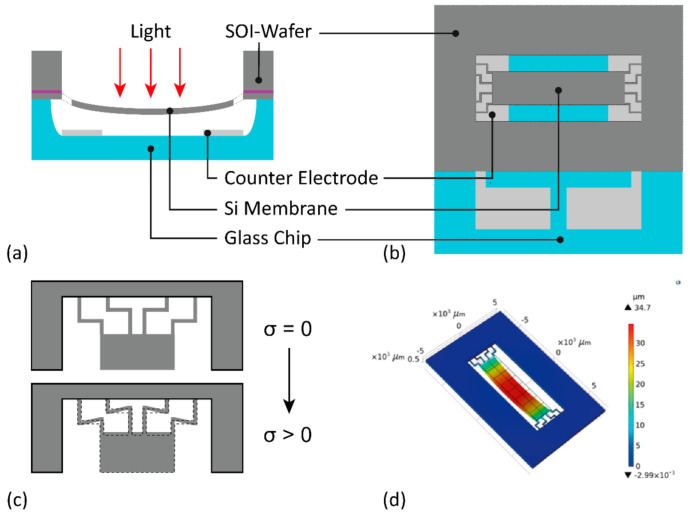
Schematic cross section (**a**) and top view (**b**) of the MAAM mirror. An incoming collimated ray bundle is reflected to a focus defined by the parabolic profile of the Si membrane. (**c**) To avoid buckling of the reflecting membrane surface by internal residual stress in the membrane material, special beams are used as suspensions of the membrane to the rim. (**d**) FEM simulation of the electrostatically deformed MAAM showing the parabolic deformation in the long axis and a flat surface in the short axis direction; applied voltage U = 250 V, electrode distance (at U = 0 V): 60 µm.

The electromechanical design of the MAAM mirror consisted of a silicon membrane, suspended over an aluminum counter electrode, deposited on a fused silica chip (Figure 2). The membrane acts as the MAAM mirror and exhibits the desired parabolic surface curvature variation when a voltage is applied between membrane and counter electrode. The basic concept of the cylindrical mirror, in which only the long axis is deformed parabolically whereas the short axis should remain as flat as possible, has been derived from a point-symmetric, round mirror design described in [26]. Two design features are important to obtain a large aperture with a purely parabolically deformed optical surface: a so-called “soft” mechanical support of the membrane at the rim, which is realized by micromachined thin and narrow beams and an electrostatic force acting predominantly at the outer part of the deformable membrane. Additionally, because stress in thin layers is a critical aspect in any kind of deformable MEMS structure, wave front errors caused by stress-induced membrane distortions (e.g., out-of-plane buckling) must be addressed for the use of reflective focusing. To this end, the membrane suspension was designed in such a way as to allow for in-plane stress release and reduce out-of-plane buckling [27].

Figure 2 schematically shows the cross section and top view of the working principle and design. The incoming collimated ray bundle is reflected to defined focus points, depending on the actual deformation state of the membrane. The focal length *f* is given by the well-known relationship:

(1) $$f\left(R\right)=\frac{1}{2}\cdot R$$

where *R* denotes the parabolic radius of the membrane deformation [28]. In contrast, ray bundles perpendicular to the parabolic profile, i.e., arranged in the plane direction of the membrane, are not focused by it.

The membrane itself is the deformable electrode. The fixed counter electrodes have a stripe-like design where the stripe length is perpendicularly oriented to the long axis of the membrane and the two stripes are placed below the two outer edges of the focusing Si-plate, which is deformed parabolically. Thus, the electrostatic force for the deformation of the membrane acts predominantly at the outer rim of the long side. To avoid buckling of the stress sensitive reflecting membrane surface by internal residual stress in the membrane material, specially formed beams are used as suspensions of the membrane to the rim in the presented MAAM. These knee-like beams allow an effective in-plane relaxation of a uniform residual stress in the membrane, as shown qualitatively in Figure 2c. This was verified by FEM simulations, showing that these beams allow an in-plane relaxation and a reduction in membrane buckling, even for a relatively large uniform intrinsic compressive stress of 10^7^ Pa inside the membrane layer material, achieving an almost completely flat surface without the application of an electric field (concave distortions less than 250 nm). In Figure 2d, the result of an FEM simulation (Comsol Multiphysics, Comsol Inc., Los Angeles, CA, USA) of the electrostatically deformed MAAM is shown. The simulated applied voltage was U = 250 V, electrode distance (at U = 0 V): 60 µm. The simulated maximum deflection, i.e., the sag of the MAAM, was about 33 µm for that design. The rectangular clear aperture of the mirror membrane had a size of 8 × 2 mm^2^. The critical design parameters for the beams are summarized in Table 1.

The micromechanical components were made from SOI wafers, and the counter electrodes were defined on a fused silica wafer into which defined cavities were etched, forming defined electrode distances. After separation from the wafer by laser dicing, the silicon membrane inside the device chip was bonded to the counter electrode chip via adhesive bonding. Details of the manufacturing process can be found in ref [29].

The MOEMS device was realized with two chips glued together with an adhesive (Norland Optical Adhesive 61, Norland Products Inc., Cranbury, NJ, USA). The device chip, holding the membrane and suspensions, was made from an SOI wafer (purchased from Si-Mat), and the fixed counter electrode was realized inside a cavity of defined depth on a fused silica wafer (from Siegert Wafer, Aachen, Germany).

**Figure 3:**
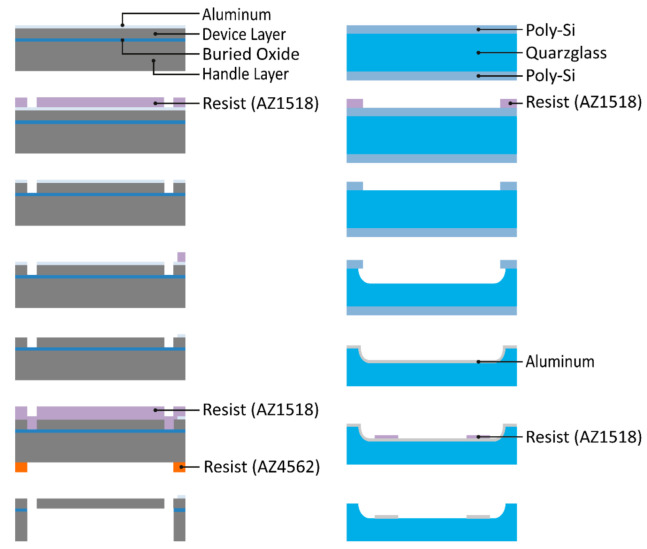
Graphical flowchart of the device chip (**left**) and the counter electrode chip (**right**).

The main steps are presented as graphical flow charts in Figure 3 for the device chip (left) and for the counter electrode chip (right). The etching steps of the device layer (defining beams and mirror geometry from the top) and of the handle layer (releasing the movable structures by etching the handle layer from the back side of the SOI) were performed by reactive ion etching (RIE) and deep reactive ion etching (DRIE), respectively, thus providing vertical sidewalls and using the buried oxide (BOX) of the SOI as the etch stop. In total, 5 lithography steps were needed (3 on the device layer, 2 on the counter electrode layer).

**Figure 4:**
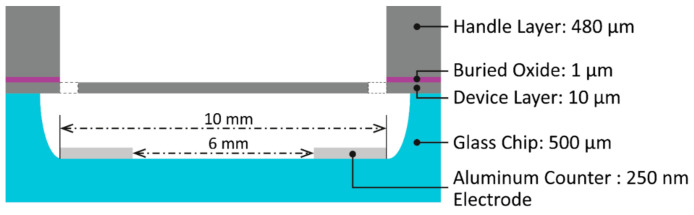
Schematic cross-sectional view of the bonded device made up of the device chip (**top**) and the glass carrier chip (**bottom**).

A schematic cross-section of the device is shown in Figure 4. The device layer chip was bonded upside down to the glass carrier chip with the counter electrodes and the cavity which defines the electrode distance and the free space to allow deformation of the membrane. The two counter electrode stripes were defined below the outer rims of the mirror and were electrically connected. The electrodes were defined in a distance of 5 and 3 mm from the mirror center (total length 8 mm). Therefore, the counter electrodes were defined also below the beams.

**Figure 5:**
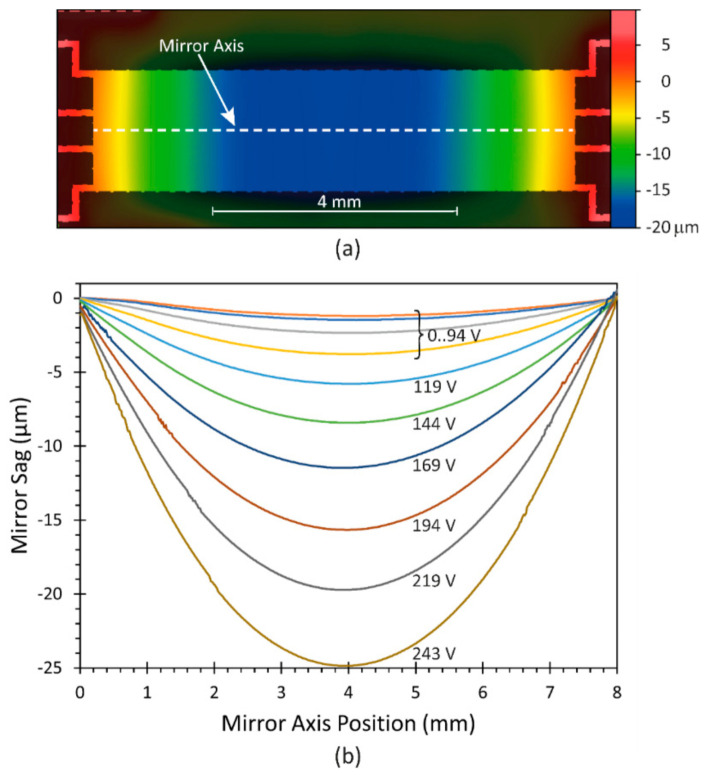
Measured deformations of the MAAM mirror for varying applied voltages: (**a**) deformation of the mirror surface and suspending structure for a membrane voltage of 219 V; (**b**) derived mirror sag measurements along the long mirror axis for voltages applied to the MAAM from 0 V to 243 V.

The membrane deformation as a function of the applied voltage between membrane and counter-electrode was measured with a White Light Interferometer (WLI)). As an example, in Figure 5a, the membrane deformation of the whole membrane for an applied voltage of 219 V is depicted. In Figure 5b the induced sag profile of the membrane along the long membrane axis is shown for voltage levels from 0 V to 243 V. The effective electrode distance was about 90 µm.

The design of the membrane should ideally create a parabolic profile along the long membrane axis; therefore, a second order polynomial fit was applied to the surface profiles. The polynomial fit function of the sag *Z* is given by Equation (2):

(2) $$Z\left(y\right)=a\cdot y^{2}+b\cdot y+c$$

where *a*, *b* and *c* denote the fit coefficients, and *y* is the position on the long mirror-axis. The fit results can be transferred into the aspheric sag equation [17]:

(3) $$Z\left(y\right)=\frac{y^{2}}{R\left(1+\sqrt{1-\left(1+k\right)\cdot \frac{y^{2}}{R^{2}}}\right)}+\alpha_{4}y^{4}+\ldots .$$

The conic constant *k* was set to −1 for a paraboloid and all aspherical parameters *α_i_* were set to 0 for *i* ≥ 4, thus the aspheric sag equation reduces to:

(4) $$Z\left(y\right)=\frac{1}{2}\cdot \frac{y^{2}}{R}.$$

Comparing Equations (2) and (4) leads to the simple relationship:

(5) $$R=\frac{1}{2\cdot a}$$

This provides the parabolic radii *R* for the optical simulations in the following section.

For clarity, the results of the polynomial fits to the measured membrane deformations in Figure 5b for the *a* parameter (see Equation (2)), the corresponding parabolic radii *R* according to Equation (5), and the resulting focal lengths *f* are already summarized here in Table 2.

**Figure 6:**
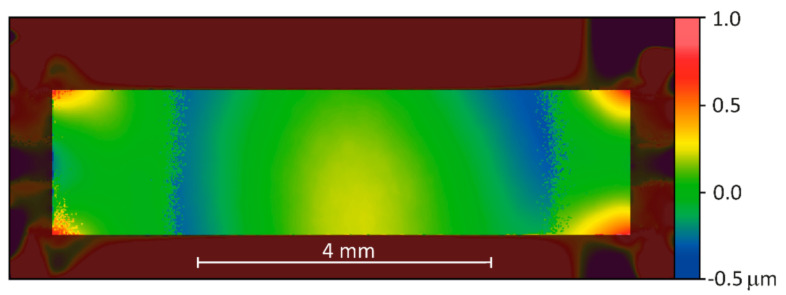
Surface deviation from an ideal parabolic cylinder surface for the deflected membrane shown in Figure 5a, membrane voltage 219 V.

In addition, to check the optical quality of the membrane mirror, the deviation of the WLI surface measurement data from an ideal parabolic cylinder surface was calculated. As an example, Figure 6 visualizes these deviation data for the deformed membrane of Figure 5a. Considering the whole membrane area, the calculated deviation ranged from −0.3 µm to +0.7 µm. Excluding the corner areas of the membrane, the deviation range reduced to −0.2 µm to +0.3 µm. The membrane comprised polished crystalline Si, therefore the surface roughness was expected to be <1 nm.

**Figure 7:**
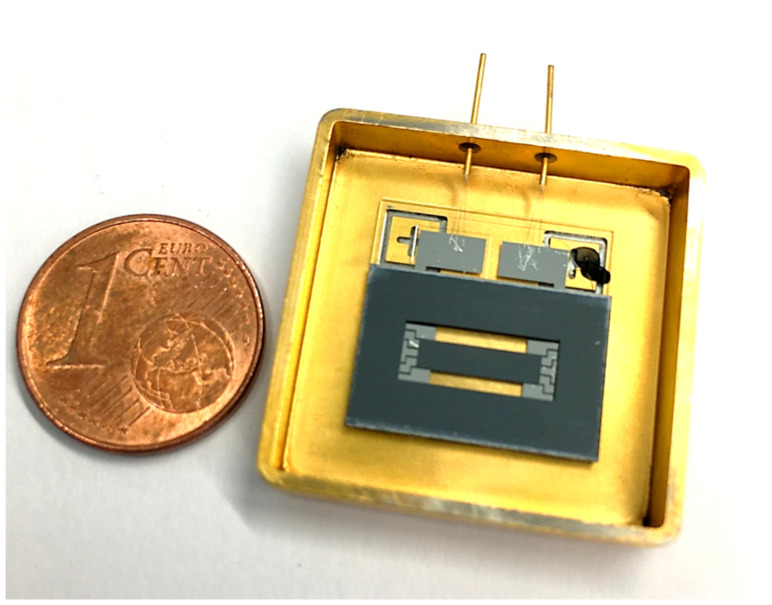
Photo of the MAAM mirror placed in a housing and bonded. The photo was taken without housing lid and glass window.

At this development stage of the MAAM mirror, the membrane itself was uncoated to avoid stress gradients in the system caused by highly stressed physical vapor deposition (PVD) coatings, such as aluminum. The mirror effect of the membrane originates from the refractive index jump between air (*n* ≈ 1) and the silicon membrane (*n* ≈ 3.85 at λ = 635 nm) with a resulting reflectivity of approximately 35%. For further protection, the MAAM mirror was placed in a housing (Figure 7) which was closed by a lid with an antireflection coated glass window; the antireflection coating was optimized for a wavelength of 635 nm and an angle of incidence of 20°.

### 2.2. Coherent System Model of the MAAM Mirror Characterization Setup

**Figure 8:**
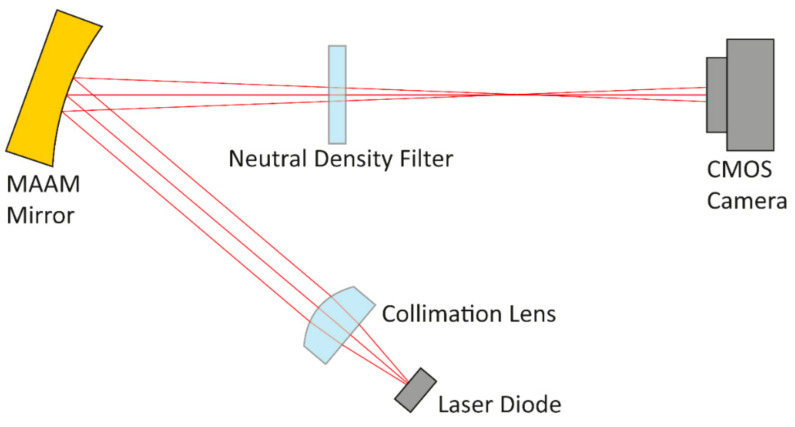
Schematic image of the MAAM mirror charaterization setup. A nearly collimated laser diode beam is reflected onto a complementary metal-oxide-semiconductor (CMOS) camera chip for beam profile characterization. The fast axis profile of the beam (within image plane) is modified by the mirror curvature. The slow axis profile of the beam (perpendicular to image plane, not shown here) remains unchanged by the MAAM mirror.

As a characterization setup, an optical system was chosen which could easily be implemented in an optical lab. A highly divergent laser diode beam was first converted into a nearly collimated beam by an aspherical lens, then directed to the MAAM mirror. The fast axis direction of the laser diode, corresponding to the long axis in the elliptical beam profile of the laser, coincided with the long axis of the membrane mirror. From there, the beam was reflected towards a beam profile detection device. The angle of incidence on the mirror was 20°, as close as the mechanical mounts of laser, lens, and mirror would allow. This helped to minimize as much as possible any off-axis aberrations caused by the curved mirror. Figure 8 schematically shows the characterization setup.

For the transfer into the simulation model, all components which might influence the detected beam profile and for which reliable mechanical and optical design models existed, were imported into an optical simulation tool (FRED [30]). This was the case for the laser diode housing and the collimation lens. The laser diode source, the lens mount aperture, the MAAM mirror, and the CMOS camera as a beam profile detection device were generated within the optical simulation tool according to the known optical specifications. Table 3 summarizes the key optical and mechanical specifications.

Because we aimed to develop a general simulation model for laser applications of the MAAM mirror, Gaussian beamlet propagation (GBP) was chosen due to its ability to represent coherent beams in astigmatic beam configurations. GBP was introduced by Arnaud and Kogelnik [23,24] as a method to propagate astigmatic Gaussian beamlets through an optical system. It is based on the idea to represent any arbitrary electromagnetic field by a certain number of Gaussian beamlets. With careful choice of beamlet size and sampling, GBP has been demonstrated to provide reliable simulation results for line lasers [20,25].

In our setup, the astigmatism in the beam mostly arose from the fast axis divergence of 32° (FWHM) of the laser diode. To determine an appropriate beamlet size and sampling, a practical approach was to observe the simulated irradiance and field phase after free space beam propagation distances which corresponded to the typical overall distances found in the setup. The quality criterion here was to obtain smooth phase profiles in all areas with significant irradiance.

**Figure 9:**
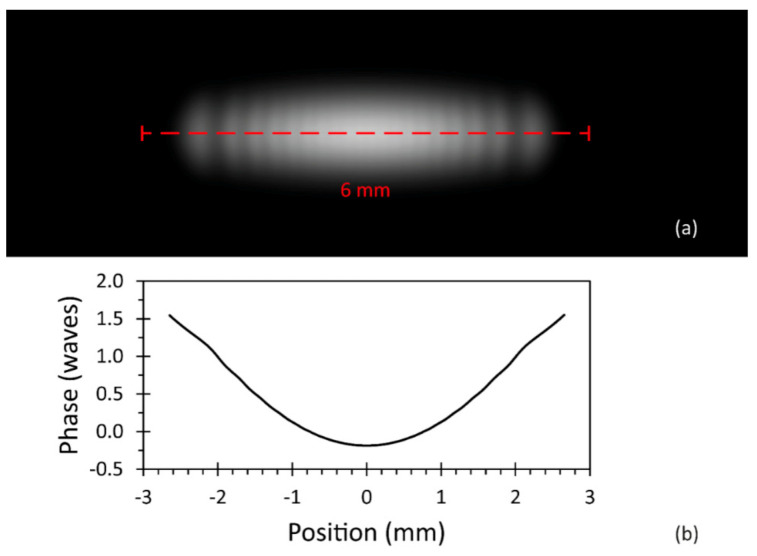
Simulated laser beam characteristics after 600 mm of free space propagation: (**a**) simulated irradiance, normalized to an image gray value of 200 for peak irradiance in the center; (**b**) simulated unwrapped phase profile along the fast beam axis.

In our case, a sampling of 2048 × 512 beamlets for the fast and slow laser diode beam axis provided reliable simulation results. As an example, Figure 9 shows the simulated irradiance and fast axis phase profile after 600 mm of free space propagation. The simulated irradiance in Figure 9a reveals a typical collimated laser diode beam profile of an elliptical shape, the ellipticity originated by the different beam divergence angles (fast axis and slow axis) of the laser diode. Along the fast axis, the irradiance exhibited a modulation, which was caused by diffraction at the collimation lens aperture. The corresponding phase of the coherent field, retrieved from the simulation and depicted in Figure 9b, showed no phase jumps, which would indicate an insufficient sampling of the coherent field. These intermediate results confirmed that the modeled laser source, consisting of the laser diode and the collimation optics, could serve as the input beam in the optical simulation for the whole system.

Finally, all component positions and orientations were implemented according to the experimental setup, which is described in detail in the next section. With this, the model was complete to simulate the laser beam profile on the detector for variable MAAM mirror radii *R* in the long axis of the membrane.

### 2.3. Experimental MAAM Mirror Characterization Setup

The experimental setup was realized according to the parameters described in the previous modelling section. In the setup, the distance between laser diode and MAAM mirror was set to 35 mm, and the distance between the MAAM mirror and CMOS camera chip to 238 mm, respectively. In order to prevent high irradiance-induced saturation effects in the camera, a neutral density filter with an optical density of 3.0 was placed between the MAAM mirror and the camera. The beam profiles changed during the focusing experiments, meaning saturation could still occur. Therefore, the exposure time of the camera was adjusted between 10 ms and 100 ms. Furthermore, the camera gain was set to a value of 0, the gamma correction to a value of 1.

**Figure 10:**
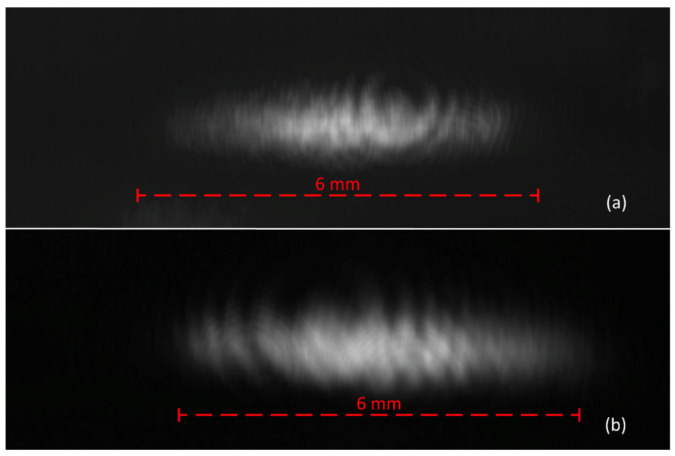
Laser beam profile measurements of the beam. (**a**) After reflection at the MAAM mirror (membrane voltage 0 V). (**b**) After 600 mm of free space propagation without MAAM. Both images are normalized to an image gray value of 200 for measured peak irradiance.

Figure 10a shows the measured beam image in the setup for the MAAM mirror at U = 0 V. The beam showed the expected elliptical shape of a nearly collimated diode laser beam but, in addition, several irradiance variations across the beam profile. These variations were more irregular, compared to the simulation of a freely propagating beam in Figure 9a. Therefore, it needed to be investigated whether these patterns had their origin in the MAAM mirror or if these patterns were the typical superimposed unintended diffraction patterns of the optical elements, dust particles, and other objects in the beam path [31,32]. For clarification, an additional image (Figure 10b) of the laser beam was taken outside the MAAM mirror setup, where the beam was propagating for just 600 mm in free space. Additionally, for the beam image in Figure 10b, taken after free space propagation, diffraction effects, similar to the ones in the MAAM mirror setup (Figure 10a), could be observed. This indicated that the patterns within the beam image observed in our MAAM mirror setup were not dominated by the MAAM mirror and originated mostly from components located before the MAAM mirror in the beam path.

### 2.4. Evaluation of Simulation and Experimental Data

The evaluation was intended to quantify the effects of the MAAM mirror on the laser beam in the current setup experimentally and by simulations. This was followed by a comparison between experimental data and simulations to validate the simulation model.

Due to the adaptive curvature in the long axis direction of the membrane, the MAAM mirror acted as a variable focusing element in this direction. The perpendicular direction of the beam should have been unaffected by the MAAM focusing device. With radii *R* ranging from 6204 mm to 327 mm (see Table 2), the corresponding focal lengths *f* in the fast beam axis varied from 3102 mm to 163.5 mm. When no voltage was applied to the MAAM mirror, the beam width in the fast axis direction was expected to be nearly the collimated beam width. In this state of the mirror, the preset focal length of 3102 mm was very large compared to the distance of 238 mm between the MAAM mirror and camera. Then, with increasing membrane voltages and correspondingly decreasing focal lengths, the focus point in the fast axis moved closer to the camera chip, leading to a decreasing beam width. Once the focal length was in the range of the camera distance, a minimum in the beam width should have been observed. Further decreasing of the focal length set the focus point of the fast axis beam in front of the camera chip, thereby leading to an increasing beam width. Due to the cylindric design of the mirror, the slow axis direction of the beam was expected to remain unchanged in width during the variation of applied voltages, radii, and focal lengths.

As can be seen in Figure 10a, the measured beam profile did not exhibit a smooth beam profile, but was superimposed by diffractive patterns. These patterns hindered the straightforward extraction of a fast axis beam width out of the measured beam profile. For example, retrieving the irradiance along a horizontal line (pixel row) followed by fitting to a theoretical function led to strong variations in the width parameter of the fit function, depending on the position of the horizontal line. Furthermore, the diffractive patterns changed with varying membrane deformations.

The aforementioned effects required a more robust method to retrieve a fast axis beam width out of the beam profiles. No significant changes in the beam width of the slow axis were observed; therefore, we averaged the irradiances on the camera images of the beam profiles along the pixel columns. This averaging reduced the effect of the superimposed patterns, but still enabled the determination of a measure for the width in the direction perpendicular to the averaging, i.e., the fast beam axis. The resulting curve was then fitted to a Gaussian function of the type:

(6) $$Irr_{av}\left(x\right)=A\cdot e^{\frac{{\left(x-\mu \right)}^{2}}{2\cdot w^{2}}}+c$$

where $Irr_{av}$ is the column averaged irradiance value at the pixel column position *x*, *A* is the fitted amplitude, *µ* is the fitted center of the profile, and *c* is the fitted offset. The fitted width *w* could then be used as the required measure for the beam width in the fast axis direction.

**Figure 11:**
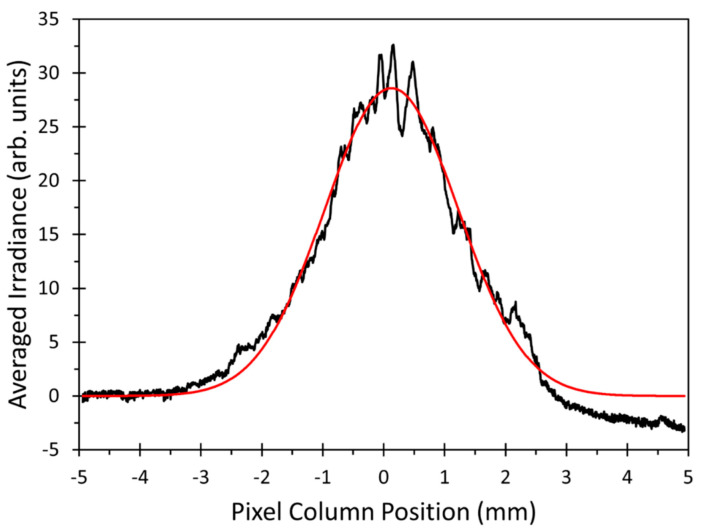
Column averaged beam profile of the laser beam in Figure 10a (black line) together with the corresponding Gaussian fit function (red line).

As an example, Figure 11 shows the pixel column averaged beam profile of Figure 10a, together with the Gaussian function and fitted according to Equation (6). The fitted Gaussian function represents the pixel column averaged irradiance in this example well enough to obtain a reliable measure for the fast axis beam width. Still, an asymmetry in the offsets at +5 mm and −5 mm of the measured averaged irradiance can be observed, which was not accounted for in the fit function. The origin of the asymmetry was not fully examined, but could be caused by stray light in the measurement setup. However, the introduction of a correction term for this offset difference in the fit function did not significantly alter the results for the beam width. Therefore, the fit function of Equation (6) was used for all measured beam profiles.

The evaluation of the results also includes the analysis of the simulation results. Therefore, the same method, averaging the irradiance over camera pixel columns, followed by fitting the resulting profile to a Gaussian function, must be applied to the simulated irradiance distribution. Grid and size of the detector cells in the simulation model were set to the parameters of the used camera; therefore, the results for the beam width *w* could be directly compared. This gave an indication on the performance of the validation method and the created model.

## 3. Results

**Figure 12:**
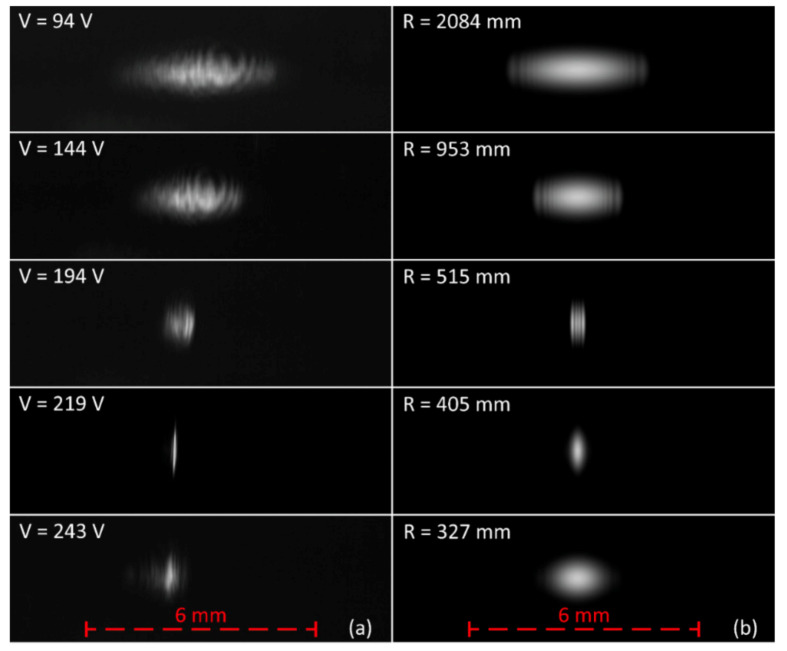
Comparison of measured (**a**) and simulated (**b**) beam profiles for varying MAAM mirror radii. For the measured beam profiles, the corresponding membrane voltages are shown in the images. For the simulated beam profiles, the mirror radii used in the simulations are shown. Note the additional simulated beam profile for the interpolated mirror radius of R = 460 mm in Figure 13, which exhibts a similar beam width like the measurement at 219 V. All images are normalized to an image gray value of 200 for the measured or simulated peak irradiance.

**Figure 13:**
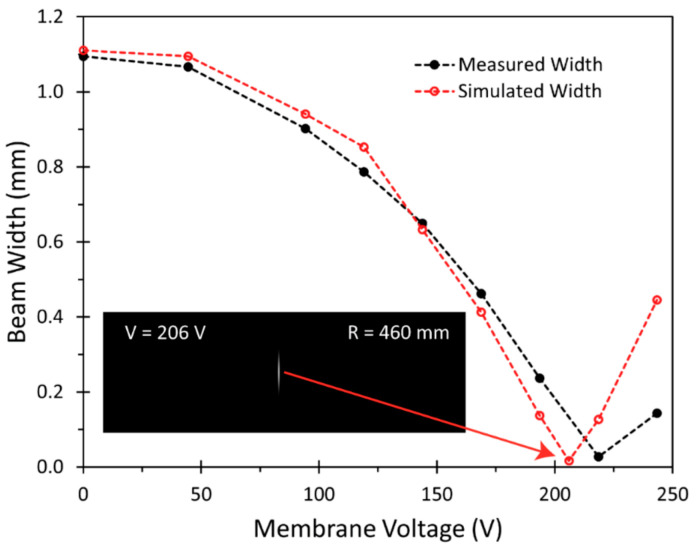
Comparison of measured and simulated beam widths *w* for varying membrane voltages. For the clarification of the trend in the simulated widths, an additional simulation point at 206 V membrane voltage, corresponding to mirror radius of 460 mm, is added. For illustration, the simulated beam profile with these parameters is inserted in the graph.

At first, the acquired beam images for varying membrane voltages were compared with the simulated beam images, calculated from the simulated irradiance data. The radii *R* used in the simulations were derived directly from the profile measurement data presented in Figure 5b, using Equations (2)–(5) and summarized in Table 2. The comparison is depicted in Figure 12, and qualitatively confirms the predicted influence of the MAAM mirror on the beam profile in our setup. In both cases, measurements and simulations, the fast axis beam width at first decreased as the nearly collimated beam was converted by the MAAM mirror into a beam with a focus point in the fast axis. Once the focus point coincided with the camera chip, the fast axis beam width reached a minimum. Further decreasing the MAAM mirror radius set the focus point in front of the camera chip, leading to an increasing fast axis beam width. Comparison of the measured (Figure 12a) and simulated (Figure 12b) beam profiles shows that the measured beam widths exhibited a smaller beam width than the simulated ones, when only the measured radii *R* of Table 2 were used for the simulations. To clarify whether a smaller beam width could be reproduced within the simulations, an additional simulation was performed with interpolated simulation parameters, *R* = 460 mm at V = 206 V (linear interpolation of *R* between values at U = 219 V and U = 194 V). The result of this additional simulation is shown as an insert in Figure 13, and confirmed that a small beam width, corresponding to the measurement, could be reproduced.

To obtain a quantitative comparison for the measured and simulated beam profile images, the resulting beam widths *w* were determined. As can be seen in Figure 13, the measured and simulated beam widths *w* satisfyingly matched over a wide range of applied voltages, derived from radii *R* for the corresponding optical simulations. The largest differences between measurements and simulations can be observed when the beam focus was close to the CMOS camera chip. One possible explanation for the behavior in this region is the strong sensitivity of the simulated beam width with respect to the membrane radii *R*. Nevertheless, the added simulation with the interpolated simulation parameters *R* = 460 mm and V = 206 V also confirms, by simulation, that a sharp beam width minimum can be achieved.

**Figure 14:**
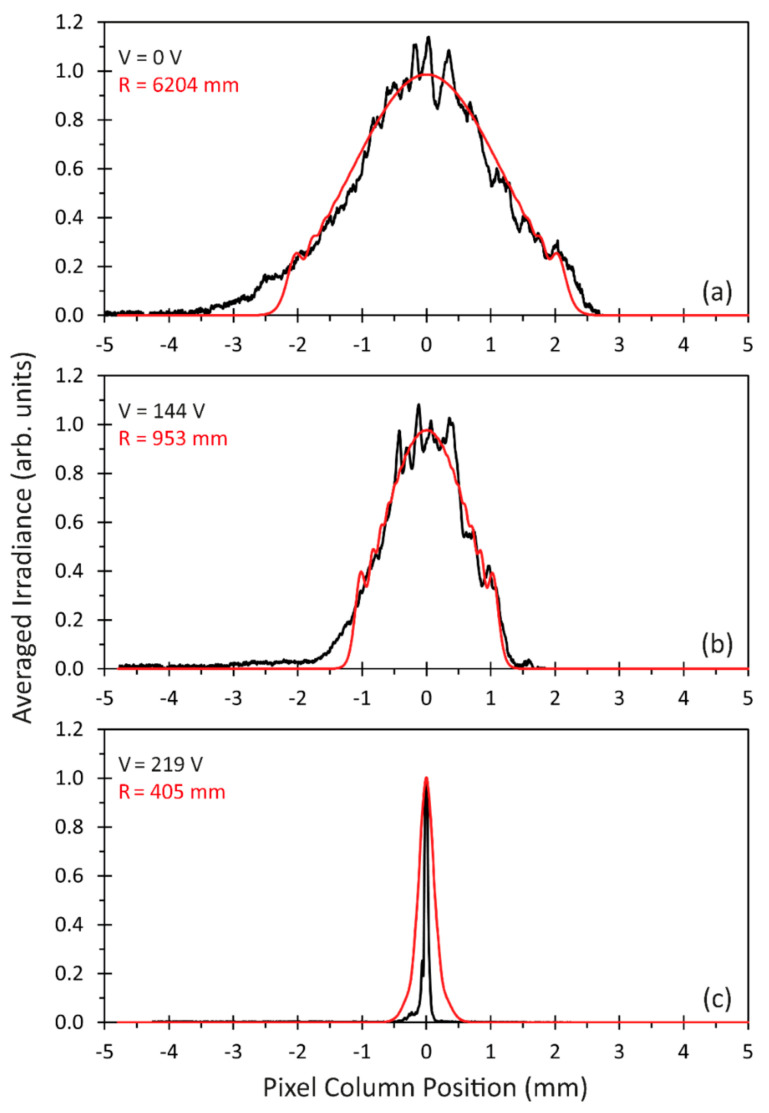
Pixel column averaged measured (black line) and simulated (red line) irradiances: (**a**) nearly collimated beam; (**b**) beam focus position behind the CMOS camera; (**c**) beam focus position near the camera chip.

Finally, the column averaged irradiance functions $Irr_{av}$ of the acquired and simulated data were directly plotted for three different cases (Figure 14).

The first case in Figure 14a represents the situation with no membrane voltage applied and the least optical power of the MAAM mirror (*R* = 6204 mm and *f* = 3102 mm, corresponding to 0.3 dpt). The second case, shown in Figure 14b, is a converging beam with the focus position behind the camera chip (*R* = 953 mm and *f* = 476.5 mm, 2.1 dpt), and the third case (Figure 14c) represents the focus position close to the camera chip (*R* = 405 mm and *f* = 202.5 mm, 4.9 dpt). The comparison between the measured data and the simulations indicates, for the first and second case, that not only do the beam widths match, but the simulated beam profiles also represent the experimental situation sufficiently well. For the third case in Figure 14c, it can be seen that the simulation provided a larger beam width. The experimental width *w*, derived from the Gaussian fit, was in this case calculated to *w* = 28 µm, whereas the simulated was calculated as *w* = 138 µm. The simulation in this case represented a situation where the beam focus lies in front of the camera chip. This is confirmed by the simulation results shown in Figure 13, with a minimum in the simulated beam width for an MAAM mirror radius *R* = 460 mm, corresponding to an interpolated membrane voltage of 206 V. For these simulation parameters, a width of *w* = 17 µm was determined. The sensitivity of the simulated beam width in this region, together with remaining measurement uncertainties and observational errors for the MAAM profiles and optical component distances, are a possible explanation for the difference between simulation and measurement in this third case.

## 4. Discussion

Overall, the accompanying simulations provided a reliable model. Due to the use of the GBP method in the simulation, this model also supports the simulation of optical systems with high beam divergence. These are the prerequisites for the development of future applications of the MAAM mirror simultaneously to the hardware improvements efforts on the MAAM mirror itself.

The results provide a first characterization of the parabolically deformable MAAM mirror as a new optical component. In its current design, it provides an optical power range from 0.3 dpt to 6.1 dpt in one direction, without introducing refractive power in the perpendicular direction. The current aperture was set to 8 mm × 2 mm, and the reflectivity was limited to 35%.

Here, future research aims to provide larger apertures and higher reflectivity. In general, with micromachining as planar technology, the planar geometries such as mirror plate size (and therefore aperture) can be simply adjusted in CAD without changes of the process conditions. This would enable the realizing of different apertures on the same wafer, and in principle provide a large degree of freedom in optical system designs using such MOEMS devices. However, with respect to aperture size, the maximal usable size is defined by the geometries (length and width), within which a perfect parabolic (long side) or flat (short side) surface is obtained within acceptable deviations (e.g., λ/5–λ/10). The surface distortion is mainly defined by internal stress in the used layers. This is especially a limiting factor when using PVD or chemical vapor deposition (CVD) layers as functional layers. For SOI, the functional layer was made from crystalline Si, and therefore theoretically stress free. However, some processes during the making of SOI wafers can introduce stress in the device layer, e.g., for the SIMOX process (Separation by IMplantation of OXygen) by implantation of oxygen or for the Smart-Cut^®^ (Soitec, Bernin, France) process by implantation of H^+^.

Therefore, a selection of appropriate SOI wafers and a better specification of stress in the device layer is needed for large, freestanding MOEMS devices providing large apertures. Additionally, large stress inside the BOX layer (in [33], a stress of −300 MPa is reported) causes an up-bending even of a thick chip rim [34]. Therefore, special developments for an appropriate bonding process are needed for large apertures. Lower distortion is expected for thicker devices layers (stiffness increases by *t^3^* with increasing membrane thickness *t*). However, this will increase the needed voltage for a given electrode distance, or will decrease the achievable diopter range.

For increasing the reflectivity, PVD-deposited aluminum as standard CMOS metallization can provide a reflectivity of about 92%. However, standard PVD processes do not allow a tight control of low stressed layers, e.g., for double side coating, and thus stress compensation. Therefore, a large aperture high-reflective MAAM will require further research on low stress, or at least well stress-controlled, reflective coatings.

In conclusion, with its MAAM mirror prototype and the accompanying optical simulation model, our research provides a sound basis for the further development of MAAM mirror-based applications and systems. Using MEMS technology, the presented MAAM can be realized at low cost in cases of high-volume production; thus, enabling its use in high-volume, price-sensitive applications.

## Figures and Tables

**Table 1:** Design parameters of the MOEMS parabolically deformable cylinder MAAM device.

Thickness of Membrane/Beams	Total Length of Beams	Width of Beams	Length of Membrane	Width of Membrane
10 µm	1.5 mm	100 µm	8 mm	2 mm

**Table 2:** MAAM parameters, obtained from surface measurements (see Figure 5) and converted into optical parameters as inputs for optical simulations.

Membrane Voltage (V)	*a* (µm/mm^2^)	*R* (mm)	*f* (mm)
0	0.0806	6204	3102
45	0.0970	5155	2578
69	0.1508	3316	1658
94	0.2399	2084	1042
119	0.3625	1379	690
144	0.5247	953	477
169	0.7236	691	346
194	0.9701	515	258
219	1.2333	405	203
243	1.5276	327	164

**Table 3:** Key specifications of the optical components in the characterization setup.

Component	Key Specifications	Manufacturer and Type
MAAM Mirror	Radius Range: 6204 mm to 327 mm Aperture: 8 mm × 2 mm Reflectivity: 35%	
Laser Diode	Optical Output Power: 3 mW Wavelength: 635 nm Beam Divergence (FWHM): 32° × 8° Housing: TO-5	Thorlabs L635P5 (Thorlabs Inc., Newton, NJ, USA)
Collimation Lens	Focal Length: 4.51 mm @ 780 nm NA: 0.55	Thorlabs C230TMD-B
CMOS camera	Type: Monochrome VIS Pixel Number: 2048 × 2048 Pixel Size: 5.5 µm × 5.5 µm	FLIR GS3-U3-41C6M-C (FLIR Systems Inc., Wilsonville, OR, USA)
Neutral Density Filter	Optical density: 3.0 Aperture: 25 mm	Thorlabs NE30A

## Data Availability

The data presented in this study are available on request from the corresponding author.
